# Intravitreal Dexamethasone Implant as a Sustained Release Drug Delivery Device for the Treatment of Ocular Diseases: A Comprehensive Review of the Literature

**DOI:** 10.3390/pharmaceutics12080703

**Published:** 2020-07-26

**Authors:** Claudio Iovino, Rodolfo Mastropasqua, Marco Lupidi, Daniela Bacherini, Marco Pellegrini, Federico Bernabei, Enrico Borrelli, Riccardo Sacconi, Adriano Carnevali, Rossella D’Aloisio, Alessio Cerquaglia, Lucia Finocchio, Andrea Govetto, Stefano Erba, Giacinto Triolo, Antonio Di Zazzo, Matteo Forlini, Aldo Vagge, Giuseppe Giannaccare

**Affiliations:** 1 Department of Surgical Sciences, Eye Clinic, University of Cagliari, 09124 Cagliari, Italy; 2 Institute of Ophthalmology, University of Modena and Reggio Emilia, 41121 Modena, Italy; rodolfo.mastropasqua@unimore.it; 3 Department of Surgical and Biomedical Sciences, Section of Ophthalmology, University of Perugia, S. Maria della Misericordia Hospital, 06129 Perugia, Italy; marco.lupidi@ospedale.perugia.it (M.L.); alessio.cerquaglia@studenti.unipg.it (A.C.); 4 Fondazione per la Macula Onlus, DINOMGI., University Eye Clinic, 16132 Genova, Italy; 5 Centre de l’Odéon, 113 Boulevard St Germain, 75006 Paris, France; 6 Department of Neurosciences, Psychology, Drug Research and Child Health, Eye Clinic, University of Florence, AOU Careggi, 50139 Florence, Italy; d.bacherini@unifi.it (D.B.); luciafinocchio@gmail.com (L.F.); 7 Ophthalmology Unit, S. Orsola-Malpighi University Hospital, University of Bologna, 40138 Bologna, Italy; marco.pellegrini9@studio.unibo.it (M.P.); federico.bernabei2@studio.unibo.it (F.B.); 8 Department of Ophthalmology, Hospital San Raffaele, University Vita Salute San Raffaele, 20132 Milan, Italy; enrico.borrelli@hsr.it (E.B.); sacconi.riccardo@hsr.it (R.S.); 9 Department of Ophthalmology, University “Magna Graecia,” 88100 Catanzaro, Italy; adrianocarnevali@unicz.it (A.C.); giuseppe.giannaccare@unicz.it (G.G.); 10 Department of Medicine and Science of Ageing, Ophthalmology Clinic, University “G. d’Annunzio” Chieti-Pescara, 66100 Chieti, Italy; rossella.daloisio@unich.it; 11 Moorfields Eye Hospital NHS Foundation Trust, London EC1V2PD, UK; 12 Fatebenefratelli-Oftalmico Hospital, ASST-Fatebenefratelli-Sacco, 63631 Milan, Italy; andrea.govetto@UHBristol.nhs.uk (A.G.); stefano.erba@asst-fbf-sacco.it (S.E.); giacinto.triolo@asst-fbf-sacco.it (G.T.); 13 Bristol Eye Hospital, University Hospitals Bristol NHS Foundation Trust, Bristol BS12LX, UK; 14 Ophthalmology Complex Operative Unit, Campus Bio Medico University Hospital, 00128, Rome, Italy; a.dizazzo@unicampus.it; 15 Domus Nova Hospital, 48121 Ravenna, Italy; matteo.forlini@iss.sm; 16 University Eye Clinic, DINOGMI, Polyclinic Hospital San Martino IRCCS, 16132 Genoa, Italy; aldo.vagge@unige.it

**Keywords:** corticosteroids, drug delivery systems, intravitreal dexamethasone implant, intravitreal injections, Ozurdex

## Abstract

Drug delivery into the vitreous chamber remains a great challenge in the pharmaceutical industry due to the complex anatomy and physiology of the eye. Intravitreal injection is the mainstream route of drug administration to the posterior segment of the eye. The purpose of this review is to assess the current literature about the widening use of the intravitreal 0.7 mg dexamethasone (Dex) implant, and to provide a comprehensive collection of all the ocular disorders that benefit from Dex administration. Although anti-vascular endothelial growth-factors (VEGFs) have been largely indicated as a first-choice level, the Dex implant represents an important treatment option, especially in selected cases, such as vitrectomized eyes or patients in whom anti-VEGF failed or are contraindicated. In this article, the safety profile as well as the list of the possible complications related to intravitreal Dex injection are also discussed.

## 1. Introduction

Intravitreal injection is nowadays considered the best accepted means of delivering drugs directly to the retina and choroid [1]. However, it is difficult to achieve and maintain significant levels of drugs into the vitreous cavity, and frequent injections are often needed.

The sustained-release of intravitreal 0.7 mg dexamethasone (Dex) implant (Ozurdex^®^, Allergan Pharmaceuticals, Irvine, CA, USA) with a biodegradable capsule of lactic acid and glycolytic acid polymers has been shown to overcome this drawback, being effective for 3–6 months [2,3].

As a corticosteroid, intravitreal Dex has been shown to suppress inflammation by inhibiting multiple inflammatory cytokines, resulting in decreased capillary leakage and the migration of inflammatory cells, edema, and fibrin deposition [4]. The United States (US) Food and Drug Administration (FDA) approved Ozurdex for the treatment of macular edema (ME), related to the following diseases: central or branch retinal vein occlusion (CRVO or BRVO, 2009), non-infectious posterior uveitis (NIU, 2010) and diabetic retinopathy (DR, 2014) [4].

**Figure 1:**
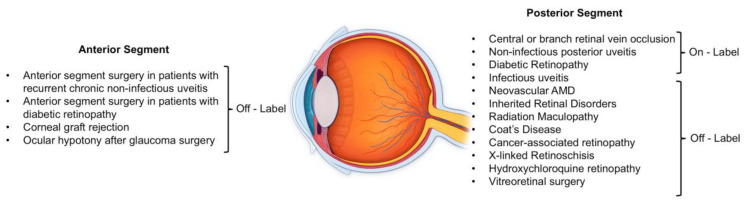
Ocular disorders that can benefit from Intravitreal Dexamethasone Injection.

To date, published reviews, articles and collected literature data about the approved use of Dex implant confirm its efficacy [4,5,6]. Nevertheless, in recent years several additional studies further confirmed the potential benefits of intravitreal Dex for various ocular pathologies with inflammatory etiopathogenesis and ME (Figure 1).

The purpose of this review is to assess the current literature about the widening use of Dex implant, and to further provide a comprehensive collection of all the ocular disorders that benefit from Dex administration. The safety profile and the list of the possible complications related to intravitreal Ozurdex injection are also discussed.

## 2. Methods

PubMed databases from January 2009 to March 2020 were searched by using the terms “dexamethasone intravitreal implant” and “Ozurdex”. Studies were limited to the English language. Registered randomized controlled studies (RCTs), prospective and retrospective, randomized and nonrandomized, single center and multicenter studies, case series and case reports were included. Anatomical and functional outcomes, as well as complications, were analyzed and discussed.

## 3. Dex Implant Mechanism of Action and Pharmacokinetic

Drug distribution in the posterior segment of the eye is a crucial step in the treatment of ocular pathologies. Dex implant contains micronized, preservative-free Dex 0.7 mg in a biodegradable copolymer of polylactic-co-glycolic acid that releases active ingredients within the vitreous chamber for up to 6 months [7]. Corticosteroids have multiple levels of action, modifying tight junction integrity, inhibiting different molecules involved in inflammation and vascular permeability process, such as Intercellular adhesion molecule-1 (ICAM-1), interleukin-6, stroma-derived factor-1, as well as vascular endothelial growth-factor (VEGF). Intravitreal Dex also targets prostaglandins and leukotrienes production blocking phospholipase A2 and paracellular permeability of Muller cells by downregulating aquaporin 4 levels [7]. A decrease of 30% in some vasoactive proteins (persephin, pentraxin 3, hepatocyte growth factor, insulin-like growth factor binding proteins, endocrine gland-VEGF) has been reported after Dex implant [7].

Dex concentrations in the retina and vitreous humor reach a plateau within days of administration and are maintained at high levels for 2 months before declining over subsequent months. A dual-phase pharmacokinetic has been described, related to the fragmentation of the implant [8]. From days 7 to 60, high concentrations of Dex in the vitreous were detected in monkeys’ eyes, whereas at days 90 to 180, Dex release was sustained at low concentrations. From days 210 to 270, Dex was below the limit of quantification [8]. Although the study was conducted in monkey eyes, the steady state concentrations of Dex achieved in monkeys are expected to be similar to those in humans [8]. Indeed, the biphasic pharmacokinetic profile resembles that obtained with the systemic pulse administration of corticosteroids and is consistent with the sustained duration of action of DEX implant seen in clinical studies. Of note, the time-point of the evaluation of Dex efficacy can account for differences in terms of functional and anatomical outcomes influencing the interpretation of the findings from different studies. Considering the Ozurdex efficacy profile, a scheduled visit at the end of the first phase is recommended to eventually consider the need for a second implant. The pharmacokinetics of 0.7 mg Ozurdex is similar in non-vitrectomized and vitrectomized eyes [9].

## 4. Dex Implant in Retinal Vein Occlusion

### 4.1. Pathogenesis of Retinal Vein Occlusion and Inflammation

Retinal vein occlusion (RVO) has been identified as the second main cause of vision loss in the working age population, following DR [10]. The known risk factors include systemic hypertension, diabetes mellitus (DM), smoking, hyperlipidemia, systemic vascular diseases, hypercoagulable states and ocular hypertension [10]. Two different types of RVO are described based on the site of occlusion: CRVO with a prevalence of 0.1–0.4% and BRVO with a prevalence of 0.6–1.1% [10].

Two main mechanisms are involved in RVO pathophysiology: (i) inner blood-retina barrier breakdown due to venous pressure rise, which is a consequence of mechanical/ischemic damage; (ii) pro-inflammatory cytokine dysregulation with consequent increase in vascular permeability and capillary network impairment [11].

Several inflammatory molecules have been identified to play an important role in the RVO pathogenesis. ICAM-1, P-selectin and E-selectin are involved in leukocyte recruitment and their adhesion to retinal blood vessel surface [10]. Moreover, high levels of VEGF and ICAM-1 in the vitreous cavity have been associated with ischemic CRVO, suggesting their role in the pathogenesis of the disease [10].

Intraretinal exudation of fluid, lipids and blood contribute to vision-threatening ME, which is a common complication of both BRVO and CRVO [12,13].

Over the years, several treatments have been proposed for ME secondary to RVO, including grid laser photocoagulation, pars plana vitrectomy (PPV), intravitreal anti-VEGF and systemic and intravitreal corticosteroids [12]. The rationale of using anti-inflammatory therapies, such as intravitreal Dex, is supported by the pathophysiology of the disease, as described above [14]. Being a water-soluble biodegradable corticosteroid with anti-inflammatory properties, Ozurdex is able to inhibit VEGF, prostaglandin and inflammatory molecule synthesis [15].

### 4.2. Evidence of the Efficacy of Dex Implant in RVO

Despite the proven efficacy of anti-VEGF in RVO resolution [16], some patients do not improve at all (20%) or even worsen (6.9%), probably due to the high variability of VEGF levels strictly related to retinal ischemia grade [17,18].

Ozurdex was approved in June 2009 by the US FDA for ME secondary to RVO, based on a phase III sham-controlled study with 6-month follow-up [19]. The GENEVA trial highlighted its beneficial effects in the treatment of 1267 patients suffering from BRVO or CVRO. In detail, 30% of patients enrolled achieved a gain of at least 15 letters visual acuity (VA) during the first 6 months of the trial compared to sham. The mean decrease in central macular thickness (CMT) was significantly higher after Dex implant as well, compared to the sham group. The GENEVA trial was extended in an open-label manner at 6 further months and, although a second implant was performed, the safety and tolerability profiles were preserved [14]. Comparable results were shown in Chinese population, where Ozurdex determined a significant improvement of functional and anatomical parameters in both BRVO and CRVO in comparison to sham [20].

Based on data collected from GENEVA trial, Danis et al. found a significant negative linear correlation between changes in CMT and in VA, with the greatest VA improvement in eyes that achieved and maintained CMT decrease at 90 and 180 days after Dex implant [21].

The SOLO study was the first multicenter trial evaluating functional and anatomical findings in patients with RVO after a unique Dex implantation with a 6-month follow-up in a real life clinical setting [22].

Early re-treatment (before 6 months) was suggested since the peak of functional and anatomical efficacy were seen at 8 weeks, with a progressive reduction in efficacy in the subsequent period. Specifically, the mean time-point of reinjection was 17.50 ± 4.20 weeks and 17.68 ± 4.20 weeks in BRVO and CRVO, respectively. Of note, in the first two months, BRVO and CRVO showed a different response to treatment with better outcomes in the BRVO group, probably related to the natural progression of the disease, which is more favorable than CRVO [22].

The onset and duration of VA improvement after Ozurdex implant in eyes with RVO have been further investigated in another study [23]. The authors reported a peak of clinical activity at 60 days (starting at 7 days), that began to decrease at 3 months [23].

A prompt treatment with intravitreal Dex in patients with RVO is associated with better clinical outcomes [24,25]. Longer ME duration at the time of first treatment was associated with significantly lower likelihood of achieving a VA gain (15 letters or more) and CMT reduction (200 micron or more) at 6-month follow-up, particularly in BRVO [25].

Ferrini et al. reported an immediate effect of the Dex implant at 24 h after the injection, while the mean time of edema relapse was identified as approximately 4.6 months [24].

In a real world setting multicenter open-label phase IV clinical study in the US (SHASTA STUDY, 26 sites, *n* = 289) patients were treated with two or more Ozurdex injections for ME secondary to RVO [26]. The mean time between two consecutive Dex implants was 5.6 months and multiple treatments demonstrated high durability and safety. A similar significant reduction in CMT was obtained between re-treatments and, as expected, intraocular pressure (IOP) increased more frequently in patients with a known history of IOP response to steroid treatment [26].

These results suggest that an accurate patient anamnesis is mandatory before starting the corticosteroid treatment. Similarly, Querques et al. reported a mean interval time of re-treatment of 4.7 ± 1.1 months and 5.1 ± 1.5 months after the first and the second implant, respectively [27].

Interestingly, the correlation between clinical improvements and angiographic findings in patients receiving intravitreal Dex for RVO, was also investigated [28]. Changes in macular leakage correlated significantly with CMT decrease in both BRVO and CRVO patients. Moreover, the proportion of eyes with active neovascularization increased from baseline to day 180 in the sham group, but remained unchanged in subjects receiving Dex injections [28].

As known, anti-VEGF agents are considered as first line treatment in eyes with RVO, although the number of injections is definitely higher and sometimes patients may not show a good clinical response [18,29].

Two multicenter RCTs investigated the efficacy and safety at 6 months of intravitreal Dex versus Ranibizumab (Rzb) 0.5 mg for the treatment of CRVO (COMRADE C study) [30] and BRVO (COMRADE B) [31].

Patients treated with Rzb showed a higher VA gain and a lower degree of retinal ischemia at 6 months [30,31]. Moreover, the results from the 1 year COMBRADE extension study provided an additional 6 months of data, revealing that BRVO and CRVO had a better response to continuous Rzb treatment in comparison to Dex in terms of VA, and this was true especially for BRVO form [32]. However, all the aforementioned studies had an important limitation because patients in the Ozurdex group were not re-treated during the 6-month follow-up [30,31,32]. As previously mentioned, Ozurdex effect peaks at 3 months and re-injection should be considered earlier than 6 months. Therefore, adopting a Dex Pro Re Nata (PRN) regimen may have provided a more robust comparison between the two treatments groups.

In a real-life study, the authors reported a significant improvement in VA with a significant reduction in CMT from baseline to week six after Dex treatment in both BRVO and CRVO [33]. Enrolled subjects were divided into three subgroups, based on ME duration. At week 12, the mean change was +9.5 Early Treatment Diabetic Retinoapthy Study (ETDRS) letters in patients with ME duration <90 days, +7.3 letters in those with ME duration between 90 and 180 days and +5.4 letters in those with ME duration >180 days. Furthermore, a very good tolerability of Dex treatment was detected in 84% of patients, while a moderate tolerability in 6% [33].

These data further support the evidence that a prompt treatment with Ozurdex in patients with RVO is associated with better clinical outcomes [24,25,33,34]. This is valid also for patients unsuccessfully treated with Rzb and aflibercept and switched to DEX [35].

A 12-month multicenter RCT (COMO study) compared anti-VEGF with multiple Dex injections in BRVO [36]. In brief, a mean of 2.5 Ozurdex injections and 8.0 Rzb injections were performed during the 12-month follow-up. The authors demonstrated that intravitreal Dex effect could be maintained only if a retreatment was performed at 4–5 months after the first implant. The use of Ozurdex was associated with an increased risk of IOP elevation and cataract progression, but a lower injection burden, compared to Rzb [36].

Of note, Kumar et al. compared the efficacy and safety of four different injection therapeutic schemes: three monthly Rzb injections (Group 1); combination of one intravitreal Rzb + laser photocoagulation (Group 2); Dex implant (Group 3); combination of one Dex implant + laser photocoagulation (Group 4) [37].

Improvements in the BCVA and CMT were comparable initially in all groups, but Rzb alone (Group 1) showed significantly higher BCVA gains at 6 months. No additional advantage of the combination with laser photocoagulation was found in both Rzb and Dex treatments [37].

In the RANIDEX study, Rzb and Dex implant were found to be both safe and effective at the 12-month follow-up in patients with ME, secondary to CRVO, with no statistically significant differences in VA and CMT changes [38].

Similar results were reported by Gado et al., who showed no significant differences at 6 months in terms of VA and CMT in patients with CRVO receiving either Ozurdex implant or bevacizumab [39].

## 5. Dex Implant in Posterior Non-Infectious Uveitis

### 5.1. Pathogenesis of Posterior Non-Infectious Uveitis

Posterior NIUs include a wide range of inflammatory conditions, affecting the uveal tissue. ME is a common complication and represents one of the leading causes of vision loss among patients affected by NIUs and its prevalence varies depending on the diagnostic technique and the underlying cause, ranging from 20% to 70% [40]. The treatment of NIUs and associated ME is still very challenging. Since inflammation is the main pathogenic event in these disorders, corticosteroids, either systemic or regional, represent the mainstay of treatment [5]. Although effective, the use of systemic corticosteroids is limited due to numerous adverse effects [41]. Intravitreal drug delivery allows us to obtain rapid and high concentrations of the drug into the eye, with a lower incidence of drug related systemic adverse events [42].

### 5.2. Evidence of the Efficacy of Dex in Posterior Non-Infectious Uveitis

Several studies showed the efficacy of Ozurdex in controlling inflammation in NIUs. The HURON trial initially investigated the safety and efficacy of a single Dex injection for the treatment of NIUs, showing a significant improvement of vitreous inflammation and VA, persisting for up to 6 months [2]. Throughout the 26-week study, the percentage of eyes in the 0.7-mg Dex implant group requiring IOP-lowering medications was 23% or less, but none needed surgical intervention for glaucoma [2].

Lightman and coauthors further confirmed the benefits of a single Dex implant in patients with NIUs for up to 6 months, reporting a significant BCVA improvement [43].

Dex implant has been proven effective and well tolerated in patients with persistent ME, resulting from uveitis or Irvine–Gass syndrome, producing significant improvements in VA and fluorescein leakage at 3 months [44].

A shorter duration of Dex implant of approximately 3 to 4 months was reported by Myung et al., albeit only four consecutive patients were included in their retrospective study [45].

A significant increase in VA and decrease in CMT and vitreous haze (VH) were observed in patients with NIUs treated with Ozurdex and followed up for at least 1 year [46]. In this study multiple injections yielded comparable visual and anatomical outcomes to single injections, and mean IOP did not change significantly during follow-up [46]. The efficacy and safety of repeated DEX implants in patients with NIUs were further confirmed by several studies, with a median duration of therapeutic effect of 6 months and a transient rise in mean IOP always well managed by topical medication [46,47,48,49].

Recent evidence suggests that Intravitreal Dex has a better safety profile and a slightly longer-lasting effect than intravitreal triamcinolone acetonide (TA) [50,51].

Of note, in a multicenter RCT it has been shown that both intravitreal TA and intravitreal Dex implants were superior to periocular TA for treating uveitic ME [52]. The risk of IOP elevation was mild and did not differ significantly between the two intravitreal treatments [52].

Significant improvements in mean CMT and VA were reported in eyes with well-controlled NIUs and persistent ME [53,54,55]. Of note, due to good clinical response, some patients reduced the daily systemic corticosteroid dosage [55]. A significant systemic corticosteroid sparing effect of the Ozurdex implant was further highlighted by Fabiani and coauthors, who reported a prompt resolution of the ME and vasculitis without any safety issue [56].

Ratra et al., investigated the role of Dex in nonresponsive ME secondary to chronic NIUs in adults and children, reporting a significant VA improvement and a good safety profile [57]. Moreover, a single injection of Ozurdex was safe and effective, as an additional treatment to systemic immunomodulatory drugs, in the treatment of refractory Behcet posterior uveitis for a 6-month period [58,59]. In a recent retrospective study, 20 patients with ocular sarcoidosis were successfully treated with intravitreal Dex injection, and only 35% of them required a second injection during the follow-up period (median 16.5 months) [60].

Ozurdex constitutes an efficacious treatment option against uveitic ME in eyes that received pars plana vitrectomy (PPV) as well, and it appears to have similar dissolution rates in vitrectomized and non-vitrectomized eyes [61]. The latter showed higher IOP increase following Dex implant than vitrectomized eyes, showing the need for close IOP monitoring [61]. Nevertheless, Dex efficacy has also been proven in challenging cases, as in a patient with NIU tamponated with silicon oil after PPV [62], and in eight patients with chronic NIU and uveitic glaucoma successfully treated with a combined surgery of Ozurdex + Ahmed drainage device [63].

Worthy of note, the Dex implant has been additionally noted to be safe and efficacious in the management of pediatric NIUs, where repeated implantations resulted in continuous control of the inflammation, allowing for the reduction in systemic immunosuppression with few systemic complications [64,65].

In 16 eyes of patients with recalcitrant Juvenile idiopathic arthritis-associated active uveitis (mean age of 17 ± 6.7), the injection of sustained-release Dex achieved the control of anterior inflammation and the resolution of ME [66].

However, further data are required to establish the safety profile of the implant in the pediatric age group [67].

DEX implant showed promising results in controlling disease activity and progression in a group of inflammatory disorders named as white dot syndromes responsible for NUI [68,69,70,71,72]. The main outcomes of these studies are summarized in Table 1.

Moreover, in two cases of bilateral idiopathic retinal vasculitis–aneurysms–neuroretinitis syndrome, the Dex implant was successfully employed to control ME in addition to panretinal photocoagulation (PRP), systemic corticosteroids, and PPV [73]; or in addition to PRP and systemic aziathioprine [74].

## 6. Dex Implant in Diabetic Retinopathy

### 6.1. Pathogenesis of Diabetic Macular Edema and Inflammation

DR represents the leading cause of visual loss and blindness in adults of working age in developed countries [75]. Central visual loss in patients affected by DR can be related to both ME and macular ischemia, with an estimated prevalence of around 20% [76].

Hyperglycemia is certainly a major cause of early microvascular changes in diabetes, causing endothelial dysfunction and the breakdown of the blood–retinal barrier.

Albeit VEGF-mediated pathways play a crucial role in DR and diabetic macular edema (DME) pathogenesis, DME shows typical features of local, low-grade chronic inflammation. Elevated levels of inflammatory markers have been detected in vitreous samples drawn from DME patients [4]. The setting of a localized, chronic and self-perpetuating inflammation is the key point of the pathogenesis of DME.

Anti-VEGF agents represent the first-line therapy for DME patients, on account of their safety and clear efficacy [77]. Nevertheless, there are patients in whom anti-VEGF drugs are contraindicated (e.g., pregnancy, high cardiovascular risk) or have demonstrated poor efficacy in improving visual function and reducing DME [78]. Therefore, intravitreal steroids, especially a sustained-release device, such as the Ozurdex implant, play a central role in the treatment of DME.

### 6.2. Evidence of the Efficacy of Dex in Diabetic Macular Edema

The Diabetic Retinopathy Clinical Research Network (DRCR.net) Protocol I study, evaluating intravitreal TA or Rzb in combination with laser treatment, demonstrated similar efficacy of both drugs in pseudophakic eyes [76]. Nevertheless, Dex has higher anti-inflammatory potency than TA and less side effects because of its higher aqueous solubility and lower lipophilicity, ensuring, therefore, a poor impact on trabecular meshwork and lens tissues [78]. The safety and efficacy of the Dex implant have been evaluated in two large, multicenter Phase III RCTs and the 3-year results of both trials were reported in the MEAD study [76,79,80]. VA improvement and macular volume reduction over the 3-year evaluation were significant with both 0.7 mg and 0.35 mg Dex (vs. sham procedure). Moreover, treatment with Ozurdex slowed the progression of DR, delaying by approximately 12 months the onset of two-step progression in Diabetic Retinopathy Disease Severity Score [79].

The safety profile of Dex implant resulted in a better safety profile compared to other intraocular steroids. In the Dex implant groups the IOP increase was usually transient, and no significant IOP changes were present after 3 years, without any cumulative effect due to repeated implants [80]. Additionally, there were no differences between the Dex arms and sham in systemic serious adverse events [80].

A subgroup analysis of the MEAD study revealed that Dex implant 0.7 mg was effective in improving VA and anatomical outcomes in the eyes of previously treated patients (with laser, anti-VEGF, TA or a combination) with a similar safety profile as compared to the overall study population [81].

Mastropasqua et al., evaluated morphological and functional changes, by means of optical coherence tomography (OCT), microperimetry and electrophysiology, in patients with DME treated with the Ozurdex implant over a follow-up of 12 months [82]. VA, macular sensitivity and central retinal thickness (CRT) substantially improved 1 month after Dex implant administration and persisted for up to 5 months. Nevertheless, there were no improvements in electro-functional parameters, which remained stable after DEX implant for up to 4 months [82].

A similar finding was reported in a short-scale prospective, multicenter, randomized study comparing the efficacy of a single Dex implant versus Dex implant, followed by retreatment on the PRN regimen over a 6-month follow-up period [83].

Lo Giudice et al., investigated the very early effect of Ozurdex implant in patients with DME [84]. More than 30% of the maximal reduction in CRT was already achieved within 3 h after injection and more than 60% within 3 days. BCVA followed at a slower rate the CRT reduction, improving significantly 7 days after treatment [84].

Interestingly, Dex implant was demonstrated to be effective even in improving other functional aspects, such as color vision. The analysis of both red-green (RG) and yellow-blue (YB) color thresholds variation after Dex implant was performed in 14 patients with DME [85]. Twenty-four weeks after injection, the RG threshold improved significantly, while YB ones remained substantially unchanged after treatment. The authors hypothesized that the DEX implant may have a direct neurotrophic effect on the retina, promoting the assessment of chromatic sensitivity as an important biomarker for monitoring DR evolution and treatment response.

Several trials were conducted in order to directly compare Ozurdex with anti-VEGFs in terms of efficacy and safety in the setting of DME [6]. In the BEVORDEX study, 42 eyes were randomized to receive bevacizumab every 4 weeks and 46 eyes were randomized to receive a Dex implant every 16 weeks in a PRN treatment regimen [86]. Patients treated with Ozurdex showed better anatomical outcomes with fewer injections (2.7 vs. 8.6). Nevertheless, these data did not translate to better VA outcomes (1% of Dex implant eyes lost 10 letters or more—mostly because of cataracts—whereas none of the 42 bevacizumab eyes did). Both drugs were able to induce a considerable regression of macular hard exudates without a statistically significant difference at 12 and 24 months between groups, albeit exudates regression seemed to be faster in Dex-treated eyes [87].

The patient-centered effectiveness of treatment, evaluated in 48 patients with the Impact of Vision Impairment Questionnaire at baseline and 24 months after, was similar in both groups (*p* > 0.1) [88]. Moreover, a post-hoc analysis of the trial highlighted how the mean retreatment interval was more than double in Dex arm compared with bevacizumab-treated eyes [89].

Ozurdex has been demonstrated to be non-inferior even to Rzb in the treatment of DME [6]. In a randomized, multicenter 12-month study, Dex implant administered every 5 months induced similar changes in VA, CRT and leakage reduction on FA when compared to Rzb [90].

Favorable results have also been reported with Dex implant in patients non-responsive to anti-VEFG or in vitrectomized eyes with DME [91,92,93,94].

In a real-world setting, eyes with DME considered refractory to anti-VEGF therapy after three monthly injections, which were switched to Dex implant, had better visual and anatomical outcomes at 12 months than those that continued treatment with anti-VEGF therapy [92,94]. In a study including 55 vitrectomized eyes, Ozurdex was found to be significantly effective in reducing CRT and vascular leakage and in improving BCVA [94].

Several trials have been conducted in order to determine if Dex implant used in combination with anti-VEGF or laser treatment could increase the anatomical and functional outcomes in persistent DME [95,96]. When compared to laser treatment alone, a significantly higher percentage of patients treated with Dex implant 1 month before laser treatment achieved at least a 10-letter improvement in VA from baseline at week 1 and at months 1 and 9 (*p* < 0.007) [95]. The CRT reduction was significantly higher in the combination group than in the laser group, and the combination treatment was well tolerated. Nevertheless, this difference was no more significant at 12 months.

Dex implant did not allow for the achievement of better visual outcomes at 12 months when compared to bevacizumab in monotherapy, whereas CRT significantly decreased in the combination group compared to the bevacizumab group [96].

Similar results have been reported for the combination therapy of Dex implant with Rzb [97]. General limitations of these trials were the confounding effect due to the lens status, which influenced the evaluation of final functional outcomes [96,97].

The rationale for combination therapy with Dex implant and anti-VEGFs has been recently strengthened by a small-size study determining the effect of both drug classes on aqueous humour cytokine expression [98]. Even though there were high inter-individual differences in cytokine concentrations in both treatment groups, monthly Rzb seemed to have a long-acting impact on VEGF and placental growth-factor levels, whereas Ozurdex showed a fast-acting action on other soluble inflammatory mediators [98].

## 7. Dex Implant in Neovascular Age-Related Macular Degeneration

### Evidence of the Efficacy of Dex in Age-Related Macular Degeneration

Age-related macular degeneration (AMD) may be complicated by the proliferation of different types of macular neovascularization. The presence of an exudative neovascular lesion characterizes the exudative or neovascular form of AMD [99,100]. Although antiangiogenic therapy is considered the gold standard of treatment for exudative neovascular AMD, previous reports have also employed corticosteroids in the treatment of these patients [101,102,103,104,105,106,107]. The use of corticosteroids is based on the assumption that inflammation is known to play a major role in the AMD pathogenesis [103,108].

A combined therapy (Dex + Rzb) may lead to an overall reduction in required Rzb injections [101,104]. Rezar-Dreindl and colleagues investigated 40 eyes with recurrent or persistent neovascular AMD [104]. Included subjects were divided into two subgroups: the first group received intravitreal Rzb with a PRN treatment regimen, while the second group received a combination of intravitreal Dex implant and Rzb. Patients receiving the combo therapy required less Rzb retreatments with consistent functional outcomes.

Conversely, Chaundhary and coauthors reported no significant advantages from the combination therapy of Dex + Rzb vs. Rzb alone [103].

The role of the Ozurdex implant was further investigated in patients with neovascular AMD resistant to anti-VEFG [105]. The authors showed significant improvements in patients receiving a single Dex injection combined with Rzb or aflibercept if compared to anti-VEGF alone [105]. These findings suggest a potential role of corticosteroids in the treatment of selected cases of neovascular AMD.

## 8. Dex Implant in Inherited Retinal Disorders

### Evidence of the Efficacy of Dex in Inherited Retinal Disorders

The use of Dex implant in patients affected by an inherited retinal disorder is usually limited to patients affected by CME secondary to retinitis pigmentosa (RP). Cystoid macular edema (CME) is a relatively common complication of RP, developing in about 20% of patients [109,110]. Table 2 summarizes characteristics and main outcomes of case reports or small series evaluating the efficacy and safety of DEX implant in RP [111,112,113,114,115].

Additionally, a couple of studies tried to elucidate the role of Dex implant in the treatment of CME secondary to RP [115,116]. Mansour et al., analyzed the outcomes of 45 eyes (34 patients) affected by CME secondary to RP treated with Ozurdex with a mean follow-up of 15.5 months [115]. The authors found that VA improved up to 4 months after the Dex implant in about 50% of cases.

Park et al., conducted a randomized, noncontrolled, paired-eye, single crossover clinical trial in which one eye of RP patients with bilateral CME was treated by Dex implant while the fellow eye was observed [116]. The authors treated 14 patients for 12 months and reported that Ozurdex could reduce the CME secondary to RP, but repeated injections were required in order to maintain the anatomical and functional results. In agreement with these results, Veritti and coauthors demonstrated that the Ozurdex implant produced better anatomic and functional outcomes in comparison to oral acetazolamide in patients affected by CME secondary to RP [117]. 

Based on these findings, intravitreal Dex implant seems to be a promising therapeutic option in patients with RP and CME. However, further prospective RCTs are warranted.

## 9. Dex Implant in Other Conditions

### Evidence of the Efficacy of Dex in Other Conditions

The intravitreal DEX implant has been successfully employed in patients with infectious uveitis, though the presence of intraocular infection represents a formal contraindication (Table 3) [118,119,120,121,122,123].

Intravitreal Dex should not be advocated as a primary treatment option in patients with infectious uveitis, however it could represent a useful and minimally invasive therapeutic resource in carefully selected cases. It can be considered a valuable adjunctive treatment, especially for patients presenting contraindication for systemic corticosteroids or requiring supplemental anti-inflammatory therapy. Several case reports and case series reported the use of the Dex implant in the management of various ocular disorders for which, at the present time, the device is not formally approved. Table 4 summarizes the characteristics and main outcomes of studies evaluating the efficacy and safety of the DEX implant in miscellaneous conditions [124,125,126,127,128,129,130,131,132,133,134,135,136,137,138,139].

## 10. Dex Implant in Ocular Surgery

### 10.1. Evidence of the Efficacy of Dex in Anterior Segment Surgery

Diabetic patients have an increased and independent risk of developing post-cataract ME, even after uncomplicated surgeries [140]. Ozurdex, combined with phacoemulsification in patients with cataract and DME, showed beneficial functional and anatomical effects for at least 3 months after surgery [141,142]. Additionally, intraoperative Dex implants performed at the time of surgery achieved the same long-term outcomes compared to a 1-month injection deferral in treating eyes with pre-existing DME [140].

Patients with NIUs have an increased risk for complications following eye surgery. These include a severe inflammatory response and unpredictably volatile postoperative IOP (ocular hypertension or hypotony).

Dex implant safely and effectively controlled postoperative inflammation in eyes with chronic recurrent NIU when concurrently implanted during anterior segment surgery, including cataract extraction or intraocular lens explantation [143]. The Ozurdex implant was also proven to be safe and effective in preventing and managing the postoperative inflammation in children with juvenile idiopathic arthritis-associated uveitic cataract [144]. Another possible application in the field of anterior segment surgery is related to immunological rejection occurring after keratoplasty. In fact, the Ozurdex implant has been shown to be an effective treatment option thanks to its potency and sustained therapeutic levels, even in cases of refractory to the standard topical and systemic therapy [145,146]. This option could be especially useful in patients with poor compliance to topical treatment, or with contraindications for the systemic therapy.

### 10.2. Evidence of the Efficacy of Dex in Vitreoretinal Surgery

Many of the pathologies requiring vitreoretinal (VR) surgery are accompanied by pro-inflammatory responses, which may result in vascular CME [147]. Retinal surgery itself may further enhance such inflammatory reactions, which may worsen or facilitate the onset of CME [148]. The use of Ozurdex as adjuvant to PPV may be useful, due to its long-lasting biological activity. This aspect may represent an advantage of Ozurdex over anti-VEGF agents, whose half-life is significantly reduced in a vitrectomized eye. Different reports have explored the safety and efficacy of Ozurdex as adjuvant to PPV in distinct retinal pathologies with a special focus on DR, rhegmatogenous and tractional retinal detachment (RD) and epiretinal membrane (ERM) [149,150,151,152,153,154].

Cakir et al., showed a prompt anatomical response to Dex implant over the first three months after PPV for CME associated with ERM [153]. With regard to surgical technique, the implant may be introduced through the same trocars used to perform PPV, at the end of the surgery. However, a 23-G trocar may be preferred as an injection through a 25-G trocar was shown to produce shattering with possible damage to the implant [155].

It may also be preferable to inject the implant when the eye is filled with balanced saline solution (BSS) rather than air, to avoid possible retinal trauma due to the implant’s kinetic energy. If needed—i.e., in the case of RD repair—fluid-air exchange may be performed after this procedure [155].

One of the main issues when using Ozurdex as adjuvant to vitrectomy is a possible rise in IOP in the postoperative period. PPV has already been shown as an independent risk factor for the new-onset of open-angle glaucoma after surgery [156,157], and the use of Ozurdex may further boost such a risk. However, this hypothesis is speculative as there is no clear evidence on this matter in the published literature.

Surgical intervention with PPV may be required in the case of proliferative DR accompanied by vitreous hemorrhage, due to neovascularization, the presence of proliferative fibrovascular complexes causing tractional RD or a combination of these factors. PPV may be indicated even in the presence of tractional CME, normally associated with tractional ERMs or vitreomacular traction (VMT).

Kim et al., found the use of Ozurdex as adjuvant to PPV particularly effective in case of tractional CME if compared to the vascular subtype [152]. In such cases, the net benefit of Ozurdex is controversial, as the action of PPV alone may be sufficient to resolve tractional CME [148]. However, a purely tractional aetiology is rare in DME and often tractional and exudative components may overlap [148].

Comparable results were obtained by Pang et al., in a retrospective report analyzing functional and anatomical outcomes of an intraoperative Dex implant during PPV for DR [158]. Moreover, Jung and Lee found PPV plus internal limiting membrane peeling and the Dex implant to be effective in improving vision and reducing retinal thickness in eyes with persistent DME [154].

ERMs may be associated with CME, whose origin can be either tractional, exudative, or a combination of both [148]. In such contexts, the anti-inflammatory action of Ozurdex may be synergic with the mechanical release of tractional forces achieved with PPV.

Iovino et al., reported favorable anatomical and functional outcomes of the Dex implant as adjuvant to PPV in advanced stage ERMs with CME [150]. In this study, both visual recovery and reduction in macular thickness were higher in the Ozurdex-treated cohort up to 6 months after surgery, if compared to the non-Ozurdex treated control group. Similar results were illustrated by Hostovsky in a separate report [151].

Worthy of note, microcystoid ME does not respond to steroids as the pathophysiology of this condition is not inflammatory [159]. Further, as this entity may be found in eyes with advanced glaucoma, the use of Ozurdex may be contraindicated due to the risk of further increase in the IOP.

The use of the Dex implant in complex rhegmatogeneous and tractional RD associated with proliferative vitreoretinopathy (PVR) was explored by a few reports, and its use is controversial, as no consensus has been reached [149,160]. Based on the hypothesis that PVR pathophysiology is mainly inflammatory, steroids such as TA or Dex have been considered promising as prophylaxis and/or treatment options [147].

A prospective, RCT analyzing 140 patients undergoing PPV for PVR detachments did not find any difference in the postoperative anatomical and functional outcome between eyes with or without Ozurdex as adjuvant to PPV [149]. However, a reduction in CME rate was observed in eyes treated with Ozurdex. Conversely, Iglicki et al., described favorable outcomes in eyes with diabetic tractional RD treated with PPV and Ozurdex [160]. CME can complicate the postoperative recovery of RD repair with either PPV or scleral buckling (SB). Risk factors for postoperative CME in vitrectomized eyes include a greater number of surgeries, higher rates of PVR, and higher rates of retinectomies [161]. Few reports confirmed the efficacy of Ozurdex in post-surgery CME in patients receiving PPV [162], and SB surgeries [163].

## 11. Safety Profile of Dex Implant

Although IOP increase and cataract progression are known side effects of Ozurdex implant, some other less common events have been reported following the injection of this intravitreal drug. Hereafter, we summarize the safety profile and list the possible complications related to intravitreal Dex affecting either the anterior or the posterior segment of the eye and the adnexa.

### 11.1. Dex Implant and IOP

Dex, fluocinolone acetonide, and TA have been shown to activate different patterns of gene expression in human trabecular meshwork cell lines [164]. However, Dex differs from TA in pharmacologic activity, lipid solubility and delivery requirements. It is less lipophilic and does not accumulate to the same extent in the trabecular meshwork, with a lower risk of IOP increase [164]. Most commonly, the IOP increase after Ozurdex injection is typically noticed within the first 2 weeks, peaks roughly at day 60 and starts decreasing gradually to get to pre-injection values usually within 180 days [165]. No cumulative effect in IOP rise is reported, and no correlation is observed between glaucoma progression and number of DEX implants [165]. IOP rises greater than 10 mmHg were detected in 24–27% of the patients enrolled in the MEAD study and were managed with IOP-lowering medications [80]. During the follow-up period, only 1 patient needed filtration surgery to control the IOP.

On the other hand, the ARTES study group reported IOP values higher than 25 mmHg and 35 mmHg in 7.9% and 0.9% of the eyes with the Dex implant, respectively [166]. Again, all these cases were managed with anti-glaucoma medications, without the need of filtering surgery. Noticeably, glaucoma patients seem to be at higher risk of IOP spikes following Dex implant, supposedly because of an already impaired trabecular meshwork aqueous outflow before the Ozurdex injection. Nevertheless, the concomitant presence of glaucoma itself is not an absolute contraindication to Dex implant [167].

Similarly, previous history of IOP spikes after Dex implantation is a risk factor for future IOP rises following further injections. Therefore, patients with previous diagnoses of glaucoma or steroid-response after a Dex implant, need to be monitored more closely to detect IOP spikes [167]. Other significant risk factors for IOP increase after Dex implant were identified in young age, male sex, type I DM, with a previous history of uveitis or RVO [165]. Additionally, Latin American and South Asian ethnicities showed greater IOP rises as compared to Caucasians [168].

Of note, the temporary elevation of IOP after Dex implantation managed with IOP-lowering agents did not seem to cause a significant reduction in retinal nerve fiber layer (RNFL) thickness, measured by OCT [169,170].

### 11.2. Dex Implant and Anterior Segment Complications

The migration of the Dex implant into the anterior chamber (AC) was not described in multicenter clinical trials, such as the MEAD study [80], whereas it was documented in real life case series [171,172]. The major risk factors related to the AC migration are previous cataract extraction or PPV, reduced zonular or capsular bag integrity secondary to complicated cataract surgery, aphakia, sulcus intraocular lenses (IOLs), iris- or scleral-fixated IOLs and AC IOLs [171,172]. All these conditions create a communication between the posterior and the anterior segment of the eye facilitating the migration of the implant from the vitreous cavity to the AC. Corneal edema and endothelial damage due to mechanical and chemical toxicity may result from the AC migration of the Dex implant [171,172]. Urgent removal of the implant is suggested to avoid corneal decompensation, permanent visual loss and the need of subsequent corneal transplantation. Several surgical techniques have been described for this purpose [171,172].

With regard to cataract formation, it has been reported that repeated DEX implant injections increase the risk of cataract progression. The European DME registrar study (ARTES) registered 12.4% of patients treated with Ozurdex implant requiring cataract surgery during the follow-up [166].

Interestingly, sporadic errors in the intravitreal injection technique led to incomplete penetration of the device in the vitreous cavity. Although this complication should be avoided at any cost, the devices reabsorbed in a couple of months [173].

Similarly, Ozurdex can be inadvertently misplaced in the crystalline lens causing a cataract. This event could lead to subsequent complicated cataract surgery if the capsular bag was broken or severely impaired, requiring iris- or scleral-fixation IOL implants [174].

### 11.3. Dex Implant and Posterior Segment Complications

Because Dex implant is delivered in the vitreous cavity, some complications related to the posterior segment deserve to be mentioned.

Overall, endophthalmitis is a major complication related to intravitreal procedures [175]. Two cases of endophthalmitis over nearly 3000 DEX implants were reported in the MEAD trial [80], while no cases of endophthalmitis were reported in the GENEVA trial [19].

A large retrospective multicenter analysis reported an endophthalmitis rate of 0.07% (two cases over 6000 injections) [165], whereas the ARTES study group reported no cases of endophthalmitis [166]. Other reported uncommon ocular side effects following Ozurdex injection are vitreous haemorrhages, rarely requiring PPV, and RD [165].

The Dex implant is linked to a higher number of secondary ERMs compared to other intravitreal steroids, such as TA. A possible explanation may be found in the poly lactic-*co*-glycolic acid (PLGA) matrix drug delivery system of the Ozurdex implant, which is biodegradable [176]. It may be speculated that PLGA may induce liquefaction of the vitreous, posterior vitreous detachment, and subsequent epiretinal response with the formation of ERM.

However, it is still under debate whether the ERM limits drug penetration into the retina [167], or it rather provokes a tractional force onto the retina itself, limiting the decrease in CMT after the injection [176].

Two cases of retinal necrosis following Ozurdex injection were published in the past years [177,178]. Retinal necrosis was caused either by Herpes Simplex Virus [177], or Cytomegalovirus [178]. Systemic steroid sparing therapy (immunosuppressive) or systemic immunodepression were recognized as the main risk factors [177,178].

Other complications of the Dex implant affecting the posterior segment are quite unusual. A case of Dex implant, adherent to the retinal surface after the filling of the vitreous cavity with gas (sulfur hexafluoride 30%) at the end of PPV, was previously described [179]. As direct contact between the Ozurdex device and the retina could cause damage to the retinal layers, the implant was promptly removed without any persistent retinal defect [179].

Two cases of Ozurdex implant desegmentation were reported without any significant intraocular complications [180].

### 11.4. Dex Implant and Adnexa

A case of Periorbital Necrotizing Fasciitis (PNF), related to Ozurdex injection, was also described [181]. The surgical trauma associated with the Dex implant was hypothesized to act as a trigger for PNF. Nevertheless, the patient had multiple risk factors, such as diabetes, old age and immunosuppressive therapy. Proper evaluation of the patients’ medical history and strict postoperative recommendations are mandatory, especially in fragile patients, to prevent such serious adverse events [181].

### 11.5. Dex Implant and Pregnancy

Anti-VEGF drugs for the treatment of retinal diseases are generally avoided during pregnancy, for the concerns related to the potential teratogen effect [182]. Steroids are considered generally safer than anti-VEGF medications in pregnant women, and they should be taken into consideration by retinal specialists. For instance, successful treatment of idiopathic and inflammatory choroidal neovascularization with Dex implant were previously reported [183].

Ozurdex may be a good option to manage the worsening of DME during pregnancy, due to the limited number of injections required to treat the condition. Published data reported both good anatomical and functional results with significant reduction in CMT and improvement of VA [184,185].

## 12. Conclusions

Intravitreal injection is the mainstream route of drug administration to treat diseases affecting the posterior segment of the eye. It is difficult to achieve and maintain significant levels of drugs into the vitreous cavity, and frequent injections are often needed [42].

Although anti-VEGFs have been largely indicated as a first-choice level, the Dex implant represents an important treatment option, especially for persistent DME, non-responders to anti-VEGF, vitrectomized eyes and in patients in whom anti-VEGF might be contraindicated (e.g., high cardiovascular risk, pregnancy) [78]. Additionally, the peculiar formulation and pharmacokinetic properties, which lead to less frequent retreatments, lower the costs and improve patient compliance, along with the satisfactory structural and functional outcomes, may allow to consider Ozurdex as a first-line approach in selected cases. Indeed, the role of DEX implant is crucial in chronic patients who often have comorbidities, requiring frequent visits to other health care professionals, since it reduces the healthcare burden both for patients and care-givers [78].

Of note, ophthalmologists should keep in mind that the type of drug, the particular design of the delivery system, and the long-lasting effect, could generate some major and minor complications. Notwithstanding, Ozurdex is still considered a safe procedure, as the most common complications reported are cataract progression and temporary IOP elevation.

In conclusion, the Dex implant is a very useful device for the management of a wide range of ocular diseases, but the choice of the best candidate to corticosteroid therapy is essential for a successful outcome.

## Figures and Tables

**Table 1:** Characteristics of studies evaluating efficacy and safety of DEX implant in White Dot Syndrome.

Author (Year)	Design	Condition	Eyes (*n*)	DEX Injections (Time Interval Range)	Follow up (Months)	Main Outcomes	Complications
Miserocchi et al. (2017) [68]	Retrospective case series	Serpiginous Choroiditis	8	Single (5 eyes), Repeated (3 eyes; 5–8 months)	18	Stabilization of serpiginous lesions, Decrease in systemic corticosteroids	Transient IOP increase (3 eyes), Cataract progression (2 eyes)
Mora-Cantallops et al. (2019) [72]	Case report	Acute Posterior Multifocal Placoid Pigment Epitheliopathy	1	Single	6	Stabilization of inflammatory lesions, Increased BCVA	Transient IOP increase
Barnes et al. (2018) [71]	Retrospective case series	Acute Zonal Occult Outer Retinopathy	6	Single	14–63	Stabilization of inflammatory lesions, Increased BCVA	Transient IOP increase, Cataract progression
Walsh et al. (2017) [69]	Retrospective case series	Birdshot Chorioretinopathy	6	Single (2 eyes), Repeated (4 eyes; 4–6 months)	12–36	Stabilization of inflammatory lesions, Reduced macular edema, Increased BCVA	IOP increase (2 eyes)
Bajwa et al. (2018) [70]	Retrospective case series	Birdshot Chorioretinopathy	6	Single (2 eyes), Repeated (4 eyes; 4–6 months)	12–36	Stabilization of the disease (2 eyes), Increased BCVA	IOP increase (2 eyes), Cataract progression (3 eyes)

DEX: Dexamethasone intravitreal implant, IOP: Intraocular pressure, BCVA: Best corrected visual acuity.

**Table 2:** Characteristics of retrospective studies evaluating efficacy and safety of DEX implant in Retinitis Pigmentosa.

Author (Year)	Design	Clinical Condition	Eyes (*n*)	DEX Injection	Follow up (Months)	Main Outcomes	Complications
Srour (2013) [111]	Retrospective case series	ME in RP	4	Single (2 eyes), Repeated (2 eyes, after 3 months)	6	Improved BCVA, Reduced CFT	None
Ahn (2014) [112]	Case report	ME in RP	2	Single	6	Improved BCVA, Reduced CFT	None
Ornek (2016) [113]	Case report	ME in RP	2	Single	6	Improved BCVA, Resolution of ME	None
Saatci (2013) [114]	Case report	ME in RP	2	Repeated after 3 months	7	Improved BCVA, Reduced CFT	None
Mansour (2018) [115]	Retrospective case series	ME in RP	45	Repeated (range 2–14 months)	Variable (15.5 ± 13.0)	Improved BCVA, Reduced CFT	Transient IOP elevation in 20 eyes, Cataract in 7 eyes

BCVA: Best corrected visual acuity, CFT: central foveal thickness, IOP: Intraocular pressure, ME: macular edema, RP: retinitis Pigmentosa.

**Table 3:** Characteristics of studies evaluating the efficacy and safety of DEX implant in Infectious Uveitis.

Author (Year)	Design	Etiology	Eyes (*n*)	DEX Injections	Follow up (Months)	Main Outcomes	Complications
Fonollosa (2016) [118]	Retrospective case series	Herpes simplex virus-type 1, Varicella-Zoster virus, Treponema Pallidum, Brucella Mellitensis, Borrelia Burgdorferi, Toxoplasma Gondii, Cytomegalovirus	8	Repeated (except 2 eyes)	6–31	Resolved ME, Improved BCVA, No reactivation of infectious disease	Transient IOP increase (1 eye)
Agarwal (2018) [119]	Retrospective case series	Mycobacterium tuberculosis	19	Single	3–4	Decreased ME, vitritis and progression of choroiditis lesions	Transient IOP increase (4 eyes), Cataract progression (2 eyes)
Jain (2018) [120]	Retrospective case series	Mycobacterium tuberculosis	9	Repeated	6–24	Stabilization of inflammatory lesions, Increased BCVA	IOP increase (2 eyes)
Lautredou (2018) [121]	Case report	Treponema pallidum and HIV	1	Repeated	15	Resolved ME, Improved BCVA	None
Majumder (2019) [122]	Case report	Treponema pallidum and HIV	1	Repeated	4	Resolved ME, Improved BCVA	Transient IOP increase
Majumder (2016) [123]	Retrospective case series	Herpes Virus	4	Repeated	6–24	Resolved ME, Improved BCVA, No reactivation of retinitis	None

DEX: Dexamethasone intravitreal implant, IOP: Intraocular pressure, BCVA: Best corrected visual acuity, HIV: Human immunodeficiency virus.

**Table 4:** Characteristics of studies evaluating efficacy and safety of DEX implant in miscellaneous conditions.

Author (Year)	Design	Clinical Condition	Eyes (*n*)	DEX Injection	Follow up (Months)	Main Outcomes	Complications
Frizziero (2017) [125]	Retrospective case series	Radiation Maculopathy following Iodine-125 brachytherapy	13	Single	6	Improved BCVA, Reduced CFT	None
Russo (2018) [126]	Retrospective case series	Radiation Maculopathy following plaque brachytherapy	8	Repeated	22	Improved BCVA, Reduced CFT	None
Seibel (2016) [124]	Retrospective case series	Radiation Maculopathy following proton beam therapy	5	Single	1	Stable BCVA in 80% of patients, Reduced CFT	None
Arrigo (2020) [127]	Case report	Combined CRVO and branch retinal artery occlusion	1	Single	24	Recovery of BCVA, Resolution of ME	None
Oztrurk (2015) [128]	Case report	Combined CRVO and branch retinal artery occlusion	1	Single	6	Improved BCVA, Resolution of ME	None
Fenicia (2013) [129]	Case report	CRAO associated with Waldenström’s macroglobulinemia	1	Single	6	Resolution of ME	None
Georgakopoulos (2019) [130]	Case report	Bilateral CRAO associated with ImmunoglobulinA multiple myeloma	2	Single	9	Improved BCVA, Resolution of ME	None
Nuzzi (2017) [131]	Case report	Anterior Ischemic Optic Neuropathy	1	Single	1	Improved BCVA and visual field	None
Saatci (2018) [132]	Case report	Coats’ disease	2	Single + Photocoagulation	50	Improved BCVA, Resolution of ME	None
Kumar (2019) [133]	Case report	Coats’ disease	1	Single + Photocoagulation	4	Improved BCVA, Resolution of ME	None
Cebeci (2014) [134]	Case report	Coats’ disease associated with vasoproliferative retinal tumor	1	Single + Photodynamic Therapy	12	Improved BCVA, Resolution of exudation	Subcapsular cataract
Kong (2019) [135]	Retrospective case series	Hypotony	15	Repeated	27	Increased intraocular pressure	Vitreous hemorrhage
Ahn (2018) [136]	Case report	Hydroxychloroquine Retinopathy	1	Single	2	Improvement of ME	None
Kim (2019) [137]	Case report	Cancer-associated retinopathy	2	Repeated (every 4 months)	48	Preservation of foveal photoreceptors	None
Mukhtar (2017) [138]	Case report	Subretinal fluid associated with X-linked retinoschisis	2	Single	6	Resolution of subretinal fluid	None
Bulut (2016) [139]	Case report	Accidental Foveal Photocoagulation by Alexandrite Laser	1	Single	3	Recovery of BCVA, Resolution of ME	Transient IOP elevation

BCVA: Best corrected visual acuity, CFT: central foveal thickness, CRAO: central retinal vein occlusion, DEX: Dexamethasone intravitreal implant, IOP: Intraocular pressure, ME: macular edema.
